# Pleomorphic rhabdomyosarcoma in a young adult harboring a novel germline *MSH2* variant

**DOI:** 10.1038/s41439-022-00185-x

**Published:** 2022-03-08

**Authors:** Akimasa Tomida, Tomohiro Chiyonobu, Shinsaku Tokuda, Mitsuru Miyachi, Kyoko Murashima, Makoto Hirata, Masanori Nakagawa, Tomoko Iehara, Junya Kuroda, Koichi Takayama

**Affiliations:** 1 Genetic Counseling Unit, University Hospital, Kyoto Prefectural University of Medicine, Kyoto, Japan; 2 Department of Pediatrics, Graduate School of Medical Science, Kyoto Prefectural University of Medicine, Kyoto, Japan; 3 Department of Molecular Diagnostics and Therapeutics, Graduate School of Medical Science, Kyoto Prefectural University of Medicine, Kyoto, Japan; 4 Department of Medical Genetics, University Hospital, Kyoto Prefectural University of Medicine, Kyoto, Japan; 5 Department of Pulmonary Medicine, Graduate School of Medical Science, Kyoto Prefectural University of Medicine, Kyoto, Japan; 6 Department of Genetic Medicine and Services, National Cancer Center Hospital, Tokyo, Japan; 7 Division of Hematology and Oncology, Department of Medicine, Kyoto Prefectural University of Medicine, Kyoto, Japan; 8 Department of Cancer Genome Medical Center, University Hospital, Kyoto Prefectural University of Medicine, Kyoto, Japan

**Keywords:** Sarcoma, Cancer genetics

## Abstract

Most cases of rhabdomyosarcoma (RMS) are sporadic and not associated with the Lynch syndrome (LS) spectrum. We report a young adult patient with RMS and a family history of colorectal cancer. Comprehensive cancer genomic profiling (CGP) of his tumor revealed a likely pathogenic variant of *MSH2*, NM_000251.3:c.1741delA (p.I581Lfs*9), which was also present in his blood sample. The widespread use of CGP may reveal that RMS can be a rare manifestation of LS.

Rhabdomyosarcoma (RMS) is a malignant soft tissue sarcoma (STS) that affects both children and adults. Most cases are sporadic. RMS has been associated with genetic syndromes such as neurofibromatosis type 1 (NF1) and Li–Fraumeni, Beckwith–Wiedemann and Costello syndromes^1^. Association with Lynch syndrome (LS) is extremely rare^2–5^. Treatment of RMS relies on a multidisciplinary approach, including chemotherapy, surgery, and radiation. Although improvements in these treatments have advanced the survival outcome, the prognosis for relapsed and refractory cases remains poor with limited therapeutic options^6^. Recently, translational and clinical research in patients with sarcomas has been conducted to develop targeted therapies^7^. In Japan, comprehensive cancer genomic profiling (CGP) for advanced solid cancer patients has been widely adopted since it became covered by the national health care insurance in 2019. Patients who progress or are likely to progress with standard therapies have received CGP of tumor samples to identify potential molecular targeted therapies. Conversely, CGP may uncover germline variants associated with cancer predisposition. Herein, we report a case of RMS in a young adult with a likely pathogenic germline *MSH2* variant.

The patient was a 29-year-old male without a history of cancer. However, he had a family history of colorectal cancer, which was present in his father, paternal grandfather, and great-uncles or great-aunts (Fig. 1A). He noticed dyspnea and was admitted to the hospital. Chest computed tomography (CT) revealed a 10-cm mass on the right chest wall with dense adhesion to the inferior vena cava (IVC) and multiple lung metastases (Fig. 1B). Hematoxylin and eosin (HE) staining of the local tumor cells by core-needle biopsy revealed high-grade malignant spindle cells showing focally striking pleomorphism. Immunohistochemistry (IHC) showed positivity for several markers associated with RMS, including desmin and myogenin. Neither the *PAX3-FOXO1* nor the *PAX7-FOXO1* fusion gene was identified by real-time polymerase chain reaction (PCR)-based analysis. The patient was diagnosed with pleomorphic RMS.

**Fig. 1:**
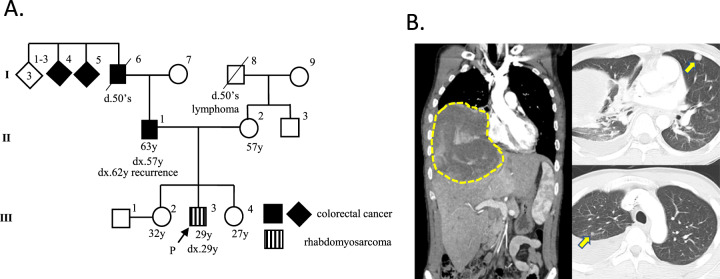
Clinical information of the patient. **A** Family tree of the patient (III-3). His father (II-1), paternal grandfather (I-6), and great-uncles or great-aunts (I-4 and I-5) had a history of colorectal cancer, which suggested a cancer predisposition syndrome similar to Lynch syndrome. P: proband, dx: age at diagnosis in years. **B** Chest computed tomography (CT) at diagnosis. The arrows show lung metastases.

He was referred to our hospital for chemoradiotherapy consisting of vincristine/actinomycin D/cyclophosphamide (VAC), ifosfamide/etoposide (IE), vincristine/doxorubicin/cyclophosphamide (VDC), and vincristine/irinotecan (VIr), according to the COG ARST0431 protocol^8^. However, the patient did not achieve complete remission. Considering potential molecular targeted therapy, we performed comprehensive genomic profiling (CGP) (FoundationOne®, Cambridge, MA, USA) of the tumor sample, which revealed variants in *MSH2* (p.I581Lfs*9), *MSH3* (p.K383Rfs*32), *NF1* (p.Y1607Lfs*15), *PALB2* (p.M296*), *CRF3R* (p.G72R), *RB1* (p.A74Efs*4), *TET2* (p.N482*) and *TP53* (p.R248Q) (Supplementary Table 1). Based on the allele frequencies, the patient’s phenotype and family history, and the potential for clinical actionability, we performed germline genetic testing for the *MSH2* variant, which confirmed a likely pathogenic germline variant in *MSH2*, NM_000251.3:c.1741delA (p.I581Lfs*9). After continuing chemotherapy, the patient underwent surgery, and the primary lesion was surgically removed. PCR-based microsatellite instability (MSI) analysis of the surgically removed tumor revealed a high MSI status. Currently, the patient is scheduled to be treated with the anti-programmed cell death 1 (PD-1) monoclonal antibody pembrolizumab after chest radiation therapy.

This patient had a family history of colorectal cancer and a likely pathogenic *MSH2* germline variant, suggesting a potential link between RMS and LS. LS is a genetic disorder associated with predisposition to colorectal cancer, endometrial cancer, and ovarian cancer. Germline mutations in mismatch repair (MMR) genes, including *MLH1, MSH2, MSH6*, and *PMS2*, are responsible for LS. Most cases of RMS are sporadic and not associated with the tumor spectrum in LS. In contrast, Ballinger et al. reported that 1.4% of patients with STSs, including RMS, have some MMR germline mutations^9^. Recently, the use of comprehensive high-throughput sequencing has led to the discovery of unexpected cancer-associated germline variants. For *MSH2*, approximately 50% of pathogenic variants identified in tumor-only sequencing are germline in origin^10^. In addition, it has been reported that germline pathogenic variants of *MSH2* are more frequently observed in STS patients with LS than are germline pathogenic variants of other MMR genes^11^.

We searched for other RMS cases related to LS in which mismatch repair deficiency (dMMR) was confirmed in both tumor tissue and blood tests. This search uncovered four more cases of RMS with LS (Table 1)^2–5^. By histology, RMS is classified into four major types: embryonal, alveolar, spindle cell/sclerosing, and pleomorphic^12^. All cases in Table 1 were of the pleomorphic type. Pleomorphic RMS has been almost exclusively found in adult patients (median age of 54 years)^13^, but the patients with both RMS and LS in Table 1 were younger (median age of 34 years). The primary site of the tumor in all four previous cases was the limbs, whereas the tumor we report here was in the chest.

**Table 1:** Rhabdomyosarcoma in Lynch syndrome patients.

						Somatic			Germline		
Case	Age sex	Histology	Location	Meta stase	MMR gene variants	MMR IHC	MSI status	MMR gene variants	Treatment	Other tumors	Tumors within third-degree family members
[2]	34 F	Pleomorphic	left upper leg	(–)	NA	MSH2 loss	high	*MSH2* (no detail information)	neoadjuvant chemotherapy surgery	HNPCC (dx.23 y)	colon cancer, thyroid cancer
[3]	50 M	Pleomorphic	right thigh	(–)	NA	MLH1/PMS2 loss	stable	*PMS2* (no detail information)	surgery adjuvant chemotherapy	HNPCC (dx.41 y)	colon cancer, pancreatic cancer, prostate cancer, melanoma, uterine cancer, thyroid cancer
[4]	36 M	Pleomorphic	lower limb	(–)	*MSH2* p.Y678*	MSH2/MSH6 loss	NA	*MSH2* p.Y678*	neoadjuvant chemotherapy radiation, surgery	None	colon cancer, thyroid cancer
[5]	19 M	Pleomorphic	right thigh	lung	*MLH1* c.1863_1864insT p.L622Sfs*10	MSH1/PMS2 loss	indeterminate	*MLH1* c.1863_1864insT p.L622Sfs*10	neoadjuvant chemotherapy surgery, anti-PD-1 antibody	None	lymphoma, colon cancer, uncharacterised cancer
This case	29 M	Pleomorphic	right chest wall	lung	*MSH2* c.1741delA p.I581Lfs*9	NA	high	*MSH2* c.1741delA p.I581Lfs*9	neoadjuvant chemotherapy radiation, surgery	None	colon cancer, lymphoma

*MMR* mismatch repair, *MSI* microsatellite instability, *IHC* immunohistochemistry, *NA* not available, *HNPCC* hereditary nonpolyposis colorectal cancer, *dx* age at diagnosis in years.

As shown in Table 1, three cases did not have an LS-related cancer history. In contrast, although dMMR was observed in all cases, only two cases had high MSI. Usually, dMMR tumors display high levels of MSI^14^. However, in STSs, pathogenic germline variants in MMR genes often do not result in high MSI^15^. Cranmer et al. reported a case of an RMS patient with a history of colorectal cancer^3^. In their case, although both colorectal cancer and RMS samples showed dMMR, the MSI-high status was detected only in the colorectal cancer samples.

Traditionally, MMR status has been determined by either PCR-based MSI testing or MMR IHC. In colon cancer, both methods have high sensitivity and concordance and can be considered equally proficient screening tests for LS^16,17^. However, to our knowledge, there are no useful reports of the concordance rate between the MSI test and MMR IHC in STS. Currently, MSI determination often relies on an alternative method, next-generation sequencing (NGS) of the tumor sample. Moreover, dMMR/MSI-high tumors were recently shown to exhibit enhanced sensitivity to immune checkpoint inhibitors (ICIs), such as PD-1 and programmed cell death ligand 1 (PD-L1) inhibitors^18,19^. ICIs have already been successfully applied to various tumors, such as endometrial cancer, stomach cancer, and colon cancer, with high MSI. A reported case with pleomorphic RMS and an intermediately elevated MSIsensor score showed complete remission with anti-PD-1 monoclonal antibody therapy following chemotherapy and surgery^5^. Our patient also underwent chemotherapy and surgery, after which the MSI-high status was revealed in the excised primary tumor. Hence, he will subsequently receive ICI therapy, which could be effective for him. However, in most atypical LS-related tumors, such as RMS, screening with MSI testing alone may yield false negative results. As a result, ICI treatment could be overlooked in some cases. We emphasize that it is necessary to document more cases of not only LS-related tumors but also atypical LS-related tumors and to further investigate the concordance between the MSI test and MMR IHC. In the future, we hope that the prescription of ICIs based on the status of MMR or the evaluation of PD-L1 by IHC of tumor samples will be applied in Japan.

In summary, this case report suggests that RMS could be a rare manifestation of LS and highlights the clinical and genetic characteristics of RMS associated with LS. The pleomorphic type is also one of the characteristics of RMS in LS. The widespread use of CGP may broaden the clinical spectrum of cancer predisposition syndromes, including LS.

## HGV database

The relevant data from this Data Report are hosted at the Human Genome Variation Database at 10.6084/m9.figshare.hgv.3134.

## Supplementary information

Supplementary Table 1

## References

[1] Dasgupta R, Fuchs J, Rodeberg D (2016). Rhabdomyosarcoma. Semin. Pediatr. Surg..

[2] den Bakker MA, Seynaeve C, Kliffen M, Dinjens WNM (2003). Microsatellite instability in a pleomorphic rhabdomyosarcoma in a patient with hereditary non-polyposis colorectal cancer. Histopathology.

[3] Cranmer LD, Chen CC, Morgan S, Martino G, Ray J (2013). Pleomorphic rhabdomyosarcoma in a patient with hereditary nonpolyposis colorectal cancer. J. Clin. Oncol..

[4] Doyle LA (2019). Characteristics of mismatch repair deficiency in sarcomas. Mod. Pathol..

[5] Tlemsani C (2020). Chemoresistant pleomorphic rhabdomyosarcoma: Whole exome sequencing reveals underlying cancer predisposition and therapeutic options. J. Med. Genet..

[6] Baruchel S (2012). A phase 2 trial of trabectedin in children with recurrent rhabdomyosarcoma, Ewing sarcoma and non-rhabdomyosarcoma soft tissue sarcomas: a report from the Children’s Oncology Group. Eur. J. Cancer.

[7] Van Erp AEM, Versleijen-Jonkers YMH, Van Der Graaf WTA, Fleuren EDG (2018). Targeted therapy-based combination treatment in rhabdomyosarcoma. Mol. Cancer Ther..

[8] Weigel BJ (2016). Intensive multiagent therapy, including dose-compressed cycles of ifosfamide/etoposide and vincristine/doxorubicin/cyclophosphamide, irinotecan, and radiation, in patientswith high-risk rhabdomyosarcoma: A report from the children’s oncology group. J. Clin. Oncol..

[9] Ballinger ML (2016). Monogenic and polygenic determinants of sarcoma risk: an international genetic study. Lancet Oncol..

[10] Mandelker D (2019). Germline-focussed analysis of tumour-only sequencing: recommendations from the ESMO Precision Medicine Working Group. Ann. Oncol..

[11] de Angelis de Carvalho N (2020). Clinical and Molecular Assessment of Patients with Lynch Syndrome and Sarcomas Underpinning the Association with MSH2 Germline Pathogenic Variants. Cancers.

[12] Leiner J, Le Loarer F (2020). The current landscape of rhabdomyosarcomas: an update. Virchows Arch..

[13] Furlong MA, Mentzel T, Fanburg-Smith JC (2001). Pleomorphic rhabdomyosarcoma in adults: A clinicopathologic study of 38 cases with emphasis on morphologic variants and recent skeletal muscle-specific markers. Mod. Pathol..

[14] Giardiello FM (2014). Guidelines on genetic evaluation and management of Lynch syndrome: a consensus statement by the US Multi-Society Task Force on colorectal cancer. Gastroenterology.

[15] Latham A (2019). Microsatellite instability is associated with the presence of Lynch syndrome pan-cancer. J. Clin. Oncol..

[16] Ladabaum U, Ford JM, Martel M, Barkun AN (2015). American Gastroenterological Association Technical Review on the Diagnosis and Management of Lynch Syndrome. Gastroenterology.

[17] Loughrey MB (2021). Identifying mismatch repair-deficient colon cancer: near-perfect concordance between immunohistochemistry and microsatellite instability testing in a large, population-based series. Histopathology.

[18] Le DT (2017). Mismatch repair deficiency predicts response of solid tumors to PD-1 blockade. Science.

[19] Zhao P, Li L, Jiang X, Li Q (2019). Mismatch repair deficiency/microsatellite instability-high as a predictor for anti-PD-1/PD-L1 immunotherapy efficacy. J. Hematol. Oncol..

