# Supplementary material for: Pleomorphic rhabdomyosarcoma in a young adult harboring a novel germline MSH2 variant
Source: Hum Genome Var. 2022 Mar 8;9:8. doi: 10.1038/s41439-022-00185-x (PMC8904467; doi:10.1038/s41439-022-00185-x)
Supplement: Supplementary file 1 — Supplementary Table 1 [file 41439_2022_185_MOESM1_ESM.pptx]

## Slide 1
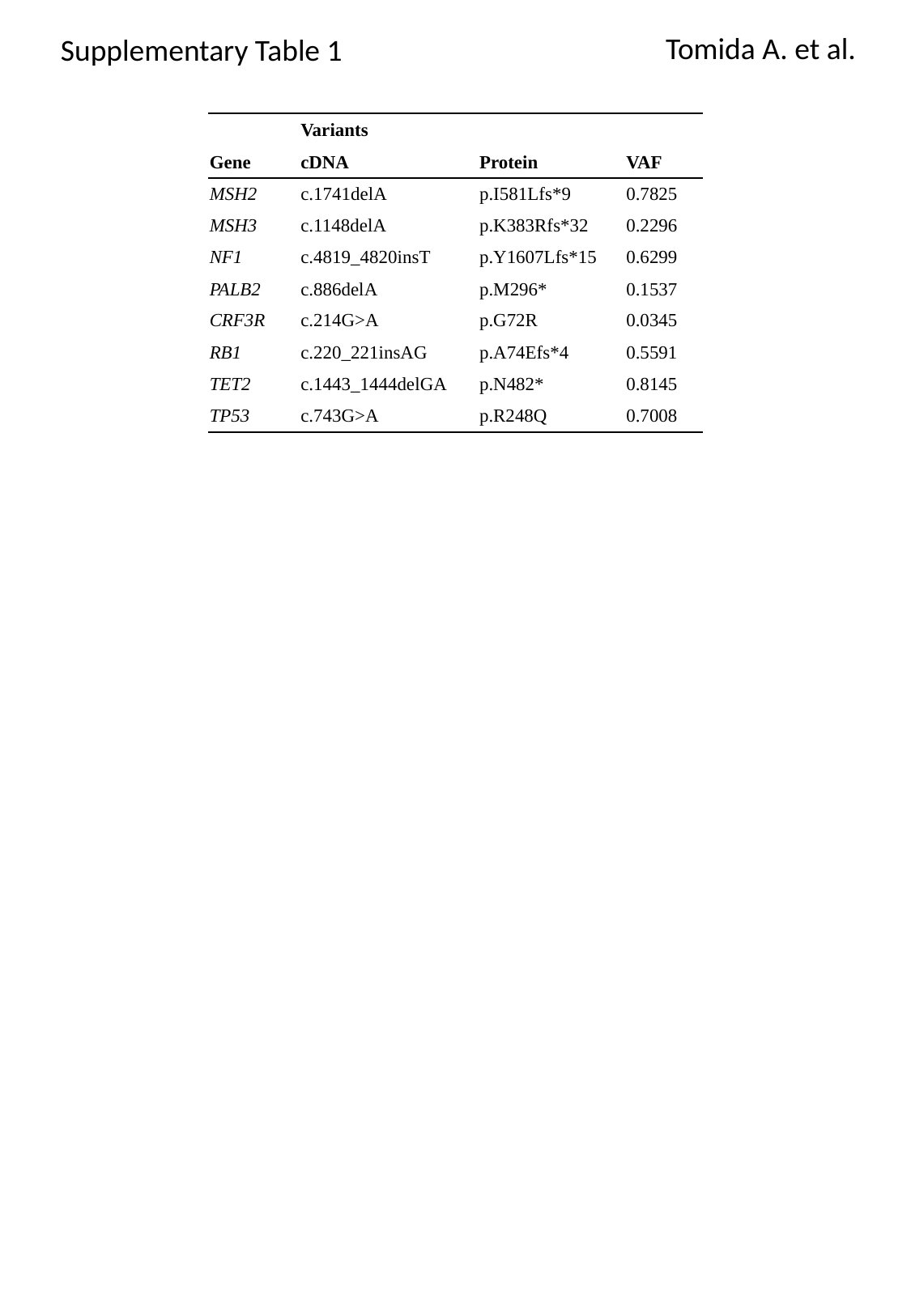

Tomida A. et al.
Supplementary Table 1
| | | | | | |
| --- | --- | --- | --- | --- | --- |
| | | | | | |
| | | Variants | | | |
| | Gene | cDNA | Protein | VAF | |
| | MSH2 | c.1741delA | p.I581Lfs\*9 | 0.7825 | |
| | MSH3 | c.1148delA | p.K383Rfs\*32 | 0.2296 | |
| | NF1 | c.4819\_4820insT | p.Y1607Lfs\*15 | 0.6299 | |
| | PALB2 | c.886delA | p.M296\* | 0.1537 | |
| | CRF3R | c.214G>A | p.G72R | 0.0345 | |
| | RB1 | c.220\_221insAG | p.A74Efs\*4 | 0.5591 | |
| | TET2 | c.1443\_1444delGA | p.N482\* | 0.8145 | |
| | TP53 | c.743G>A | p.R248Q | 0.7008 | |
| | | | | | |
| | | | | | |
